# Feasibility evaluation of radiotherapy positioning system guided by augmented reality and point cloud registration

**DOI:** 10.1002/acm2.14243

**Published:** 2024-01-16

**Authors:** Shaozhuang Zhai, Ziwen Wei, Xiaolong Wu, Ligang Xing, Jinming Yu, Junchao Qian

**Affiliations:** ^1^ School of Basic Medical Sciences Anhui Medical University Hefei P.R. China; ^2^ Anhui Province Key Laboratory of Medical Physics and Technology Institute of Health and Medical Technology Hefei Institutes of Physical Science Hefei Cancer Hospital, Chinese Academy of Sciences Hefei P.R. China; ^3^ Department of Radiation Oncology, School of Medicine, Shandong University Shandong Cancer Hospital and Institute Shandong First Medical University and Shandong Academy of Medical Sciences Jinan Shandong China

**Keywords:** augmented reality, ICP, point cloud registration, radiotherapy positioning, visualization

## Abstract

**Purpose:**

To develop a radiotherapy positioning system based on Point Cloud Registration (PCR) and Augmented Reality (AR), and to verify its feasibility.

**Methods:**

The optimal steps of PCR were investigated, and virtual positioning experiments were designed to evaluate its accuracy and speed. AR was implemented by Unity 3D and Vuforia for initial position correction, and PCR for precision registration, to achieve the proposed radiotherapy positioning system. Feasibility of the proposed system was evaluated through phantom positioning tests as well as real human positioning tests. Real human tests involved breath‐holding positioning and free‐breathing positioning tests. Evaluation metrics included 6 Degree of Freedom (DOF) deviations and Distance (D) errors. Additionally, the interaction between CBCT and the proposed system was envisaged through CBCT and optical cross‐source PCR.

**Results:**

Point‐to‐plane iterative Closest Point (ICP), statistical filtering, uniform down‐sampling, and optimal sampling ratio were determined for PCR procedure. In virtual positioning tests, a single registration took only 0.111 s, and the average D error for 15 patients was 0.015 ± 0.029 mm., Errors of phantom tests were at the sub‐millimeter level, with an average D error of 0.6 ± 0.2 mm. In the real human positioning tests, the average accuracy of breath‐holding positioning was still at the sub‐millimeter level, where the errors of X, Y and Z axes were 0.59 ± 0.12 mm, 0.54 ± 0.12 mm, and 0.52 ± 0.09 mm, and the average D error was 0.96 ± 0.22 mm. In the free‐breathing positioning, the average errors in X, Y, and Z axes were still less than 1 mm. Although the mean D error was 1.93 ± 0.36 mm, it still falls within a clinically acceptable error margin.

**Conclusion:**

The AR and PCR‐guided radiotherapy positioning system enables markerless, radiation‐free and high‐accuracy positioning, which is feasible in real‐world scenarios.

## 1 INTRODUCTION

Precision radiotherapy is characterized by more accurate target positioning and irradiation, higher and more uniform dose distribution in the target area, and less damage to the organs at risk (OAR), which requires precise radiotherapy positioning, planning and dose calculation. In contrast, inaccurate positioning can lead to over‐irradiation or under‐irradiation, decreasing the effectiveness of radiotherapy and resulting in additional radiation to normal tissues.^1^ In addition, the movement of organs and tumors due to breathing can also cause deviations in positioning, making it difficult to ensure the radiation dose to the target and enhancing the risk of radiation exposure to OAR.^2^ Hence, it is necessary to investigate and improve the radiotherapy positioning workflow to improve efficacy of radiotherapy and patient prognosis.

The laser and skin surface marker techniques are usually used in the real radiotherapy treatment, which is basically insufficient automation, inaccurate positioning and time‐consuming processes. Although the positioning accuracy could be improved with Cone‐Beam Computed Tomography (CBCT) guided method, it can cause unnecessary radiation exposure to patients and faces challenges in achieving real‐time guidance. Currently, advanced radiotherapy positioning techniques include surface‐guided radiotherapy (SGRT) and optical surface monitoring system (OSMS). SGRT relies on optical surface imaging to reduce positioning errors during treatment.^3^ OSMS employs optical methods to achieve the patient's body position information and project lasers onto the patient's body surface to realize image‐guided radiotherapy.^4^ Both of them offer advantages such as no radiation, higher precision and efficiency. However, higher costs may limit their widespread uses. Therefore, there is an urgent need to reduce the cost of radiotherapy positioning technology and minimize the errors associated with radiotherapy positioning.

In addition to the utilization of SGRT and OSMS, augmented reality (AR) has also emerged as a prevalent modality for radiotherapy positioning. For example, Perry et al. used the Hololens 2 to provide a visual reference for the positioning.^5^ Nevertheless, the method had an accuracy error of up to 3.0 ± 1.5 mm, which did not fall within the clinically acceptable error range. A previous study about AR‐assisted radiotherapy positioning systems was only conducted under optimal conditions, and the errors in all directions were above 2 mm, which posed a significant limitation.^6^ Results of AR‐assisted radiotherapy positioning in another study showed that the errors of the Y and Z axis were lower than manual positioning, but the errors were still above 2 mm.^7^ Although there are limitations of AR, it is essential that the use of AR‐guided radiotherapy positioning can achieve markerless body surface and AR visualization. AR is limited by hardware conditions such as head‐mounted devices (HMD), which may cause fatigue and poor stability for users. Consequently, we prefer to use a high‐definition (HD) camera combined with AR technology rather than HMD, which is more stable and can meet the requirements for radiotherapy positioning monitoring. In addition, the relatively low accuracy of AR suggests that we must combine it with other high‐precision technologies to meet the requirements for clinical accuracy.

Therefore, considering the current situation of radiotherapy positioning, the study aims to develop a radiotherapy positioning system assisted by AR technology combined with an HD camera for visualization and initial position correction, and 3D point cloud registration (PCR) technology for accurate alignment. To verify the feasibility of the system in real environments, system testing was conducted using a 3D surface imaging tool to provide a reference for further assisting in radiotherapy positioning, improving radiotherapy positioning accuracy, and optimizing radiotherapy quality.

## 2 MATERIALS AND METHODS

### 2.1 PCR procedure design

**FIGURE 1:**
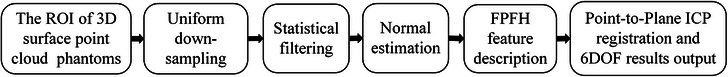
The proposed PCR procedure flow chart.

As shown in Figure 1, the PCR procedure primarily consists of 3D body surface reconstruction, PCR pre‐processing, the Point‐to‐Plane Iterative Closest Point (ICP) registration based on Fast Point Feature Histograms (FPFH), and 6 Degree of Freedom (DOF) offsets output. The details of each step are described below.

#### 2.1.1 3D point cloud reconstruction

Fifteen different patients’ CT images were selected to reconstruct surface point clouds of patients, which were subsequently utilized for PCR exploration and virtual positioning experiments. The Region of interest (ROI) of CT images needed to be fully scanned covering the area below the clavicle and above the anterior superior iliac spine. The left and right borders were required to extend up to the anterior axillary line. Due to the challenges of real‐time performance and potential radiation hazards posed by CT imaging, we chose to utilize a promising and radiation‐free optical 3D body surface imaging tool for conducting confirmatory experiments on both a human phantom and a real human subject. The ROI of the 3D surface image was consistent with the CT image reconstruction range.

Although CT imaging and optical 3D imaging rely on different imaging principles, both methods can provide body surface information. Therefore, there is no conflict in using CT data for PCR exploration and virtual positioning experiments, and using 3D scanning data for the positioning verification experiments involving both a human phantom and a real human subject. The data used in this study is retrospective patient data that has been thoroughly anonymized to the extent that individual identities cannot be identified. Therefore, informed consent is not required, and approval from the Institutional Review Board (IRB) is not necessary.

#### 2.1.2 Preprocessing of PCR

##### Uniform down‐sampling

**FIGURE 2:**
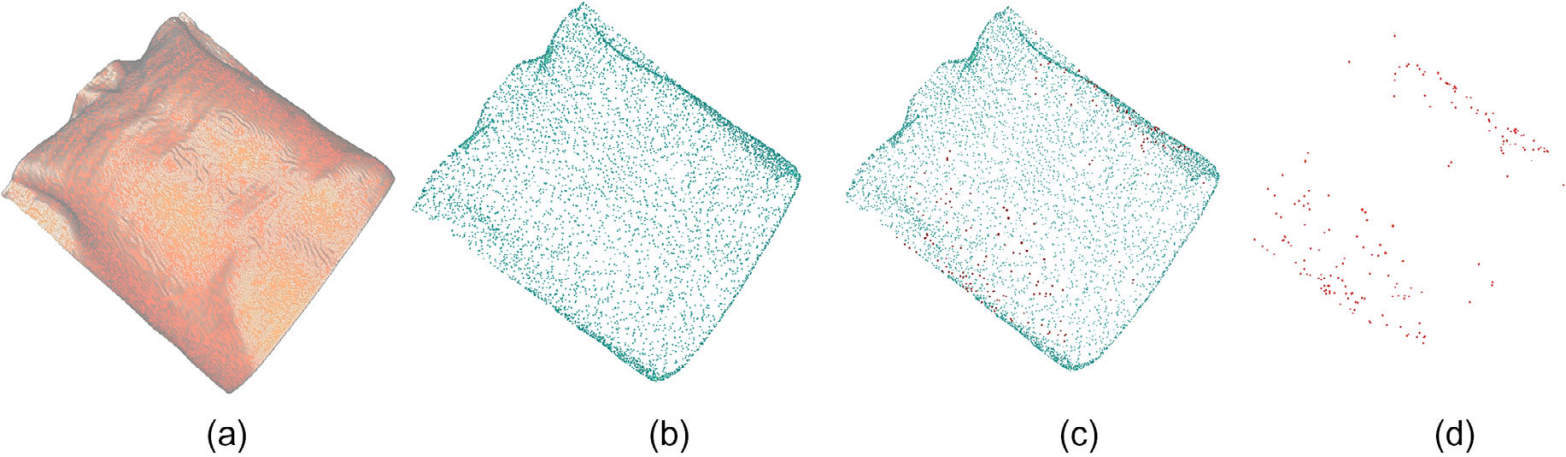
Preprocessing of the proposed PCR procedure. a: Original phantom, b: Point cloud after uniform down‐sampling, c: Statistical filter processing: red points are the discrete point identified by the filter, d: Discrete points elimination.

The down‐sampling of point cloud aimed to depress the complexity of point cloud processing by reducing the points number.^8^ Without downsampling, the surface point cloud generated from CT images contains over 120,000 points, the surface point cloud from the phantom contains over 160,000 points, and the surface point cloud from the real human body contains over 180,000 points, which could lead to registration delay. Therefore, in order to improve the registration efficiency, a uniform down‐sampling process was performed for the point cloud phantoms. The position of the reserved points was not changed after uniform down‐sampling, and the points distribution was substantially uniform,^9^ which increased the efficiency of PCR without lowering accuracy. The down‐sampling effect is shown in Figure 2b.

##### Discrete points elimination

The reconstruction of point clouds is commonly accompanied by the existence of noise, which results in registration failure.^10^ Thus we employed an outlier exclusion method to ensure the registration accuracy. The main steps involved performing statistical analysis on the neighborhood of each point and removing the discrete points.^11^ Figure 2c,d showed the recognition and elimination of discrete points.

#### 2.1.3 Point‐to‐Plane ICP registration based on FPFH

##### Normal estimation

**FIGURE 3:**
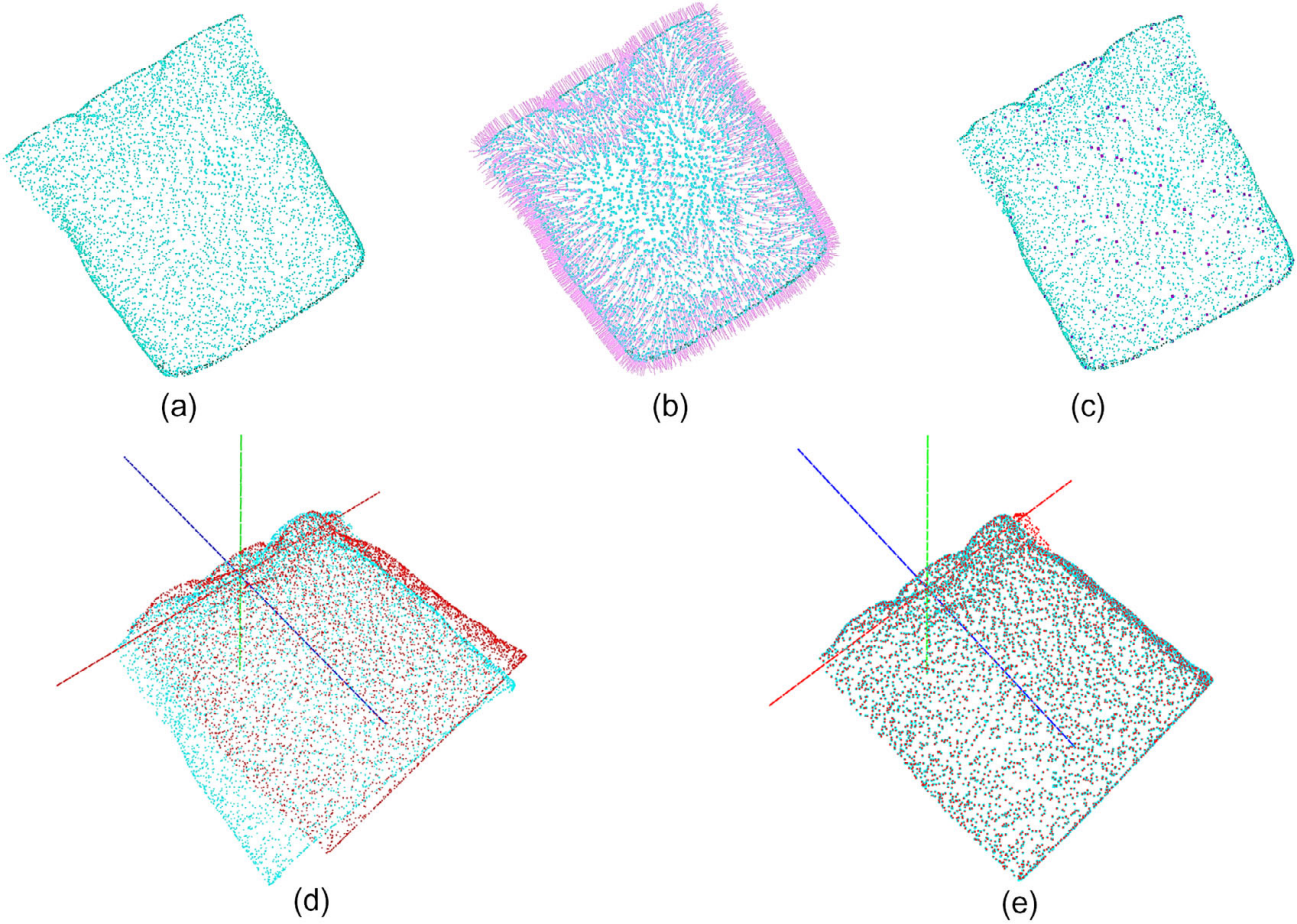
Point‐to‐Plane ICP registration based on FPFH. a: Point cloud after pre‐processing, b: Normal estimation of point clouds: the purple lines are normals, c: FPFH description: red points are the feature points, d: The two‐point clouds before ICP registration: the red point cloud is the target position, and the green is the floating position, e: Two‐point cloud with coincident positions after ICP registration.

Normal is a significant character of geometric surfaces and is heavily used for accurate light source placement and shadow reduction. The normal estimation for human surface is shown in Figure 3b. In this paper, the normal estimation serves as a necessary prerequisite to perform Point‐to‐Plane ICP registration.

##### FPFH feature points description

FPFH is a feature description based on the normal between points and their neighbors before registration.^12^, ^13^ It is more simplified and computationally faster than Point Feature Histograms (PFH). The principle of FPFH^13^ can be summarized as follows:

First, the p_q_ and its neighbors were computed to obtain a Simplified Point Feature Histogram (SPFH). Next, the k‐neighborhoods of p_q_ were redefined and the adjacent SPFH values were applied to weight the histogram of the final point p_q_, namely FPFH. The formula of FPFH was as follows:

(1) $$\mathrm{FPFH}\left(p_{q}\right)\;=\mathrm{SPFH}\left(p_{q}\right)+\frac{1}{k}\sum_{i\;=\;1}^{k}\frac{1}{\omega_{k}}\cdot \mathrm{SPFH}\left(p_{k}\right)$$

where ω_k_ was a weighting factor depending on the distance between p_q_ and neighbor p_k_ in the given metric space. In this study, the visualization of the FPFH description of the human 3D body surface point cloud was shown in Figure 3c.

##### Point‐to‐plane ICP registration

PCR is essentially the coordinate system transformation between two‐point clouds, with its main task being the computation of the rotation matrix R and translation matrix T. Usually, the ICP algorithm consists of finding the corresponding set K, extracting and updating the conversion matrix. Finally, the objective function E (T) is minimized on the corresponding set K to complete registration. The E(T) of Point‐to‐Plane ICP is shown in the following Equation (2)^14^

(2) $$E\left(T\right)\;=\sum_{\left(p,q\right)\;}{\left(\left(p-T_{q}\right)\cdot n_{p}\right)}^{2})\;\;$$

Where p and q are two‐points belonging to K and n_p_ is the normal of point p. p and q were from two‐point clouds that need to be registered, respectively.

### 2.2 AR‐and‐PCR‐guided radiotherapy positioning system design and the phantom tests

**FIGURE 4:**
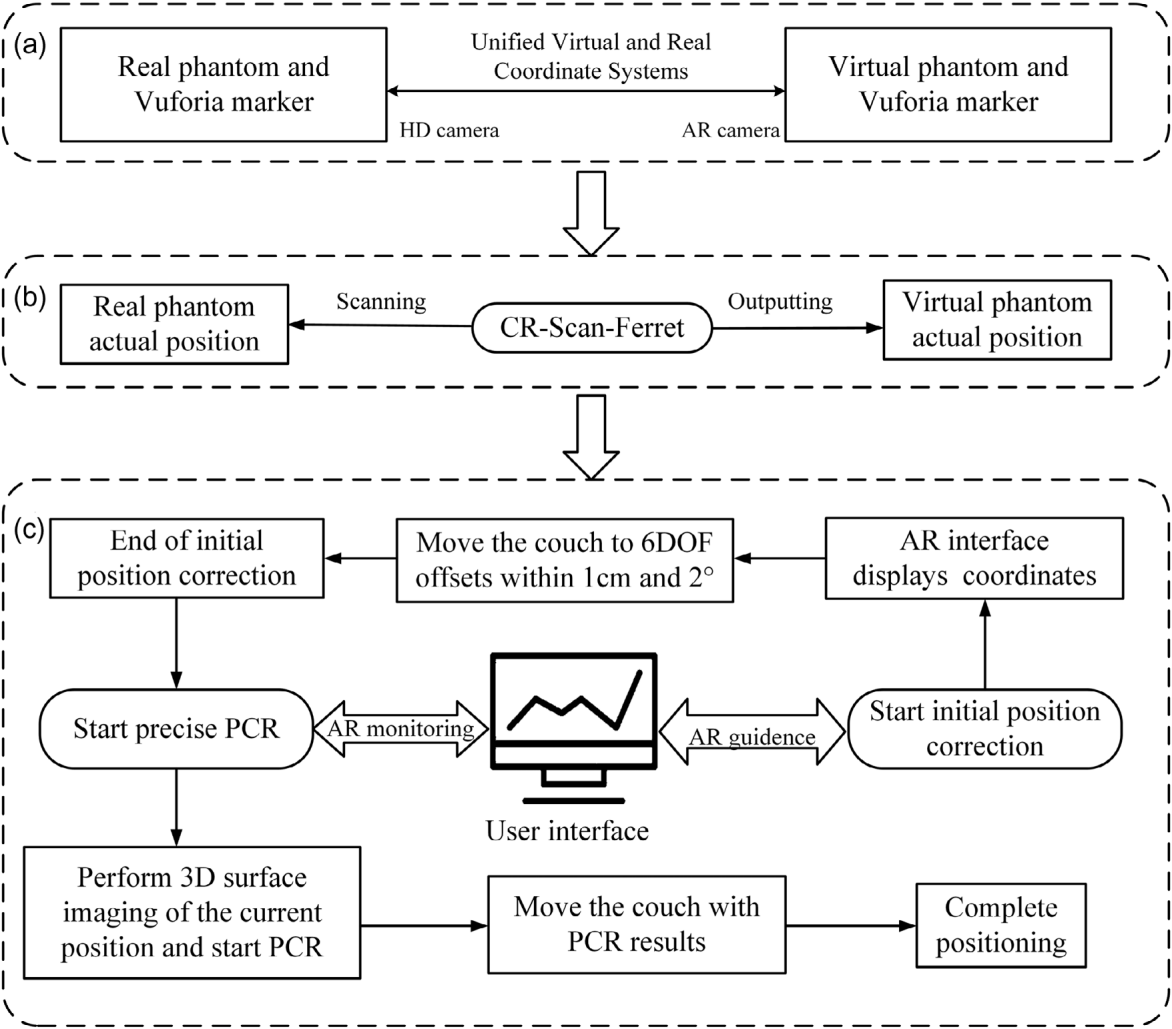
Phantoms experiment process of AR‐and‐PCR‐guided positioning system simulated radiotherapy positioning.

To utilize the aforementioned PCR system in a real environment, we combined it with AR and 3D surface imaging. Additionally, the user interface for radiotherapy positioning was designed to improve the positioning workflow using Unity 2020.3.41 and Vuforia 10.15.4, The workflow can be observed in Figure 4.

#### 2.2.1 System scenarios and devices

**FIGURE 5:**
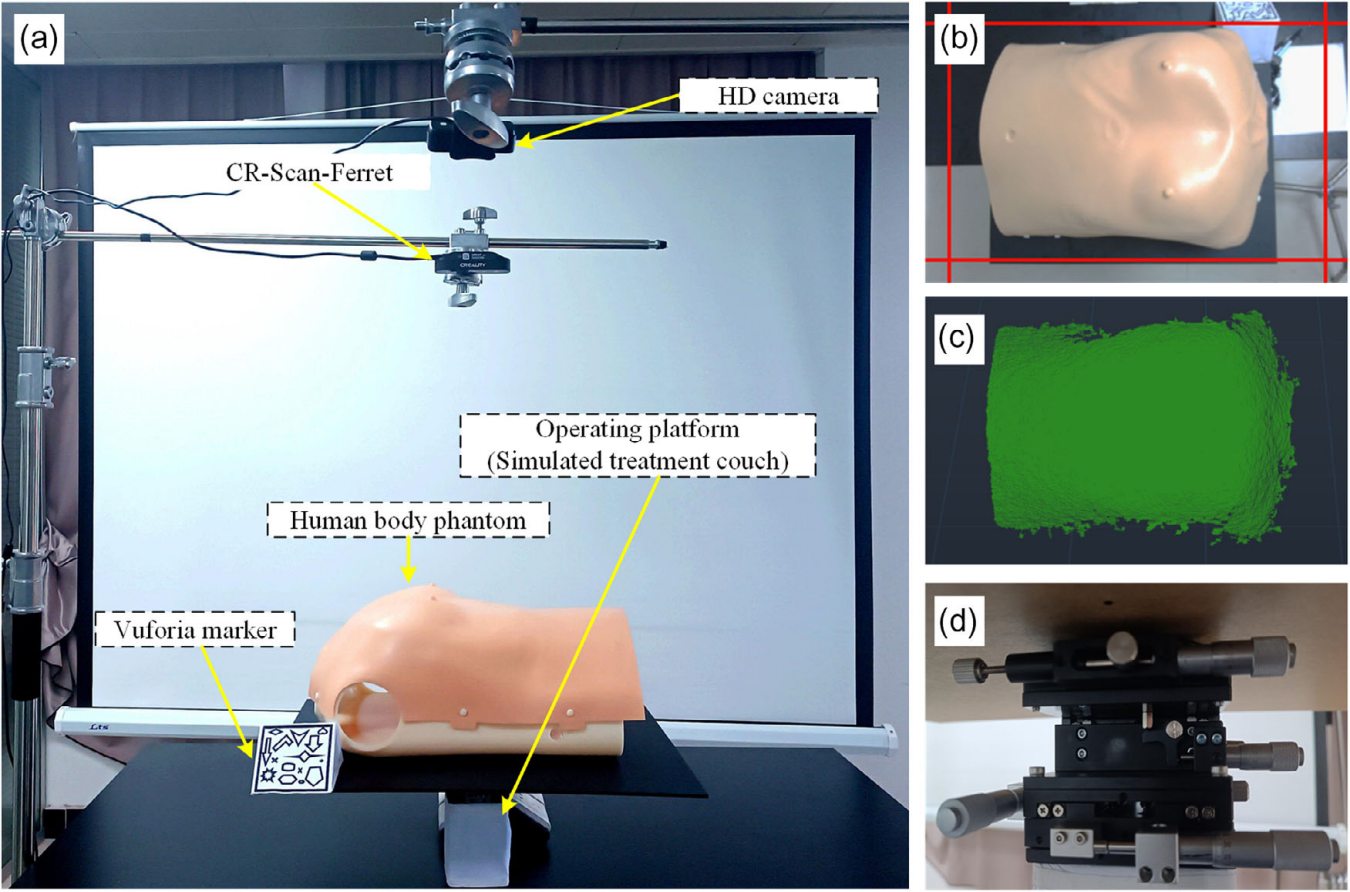
Phantom experiment scene of simulated radiotherapy positioning. a: Location of components on the experimental site, b: CR‐Scan‐Ferret scanning range, c: 3D scanning imaging, d: High‐precision operating platform (Simulated treatment couch).

The complete experimental scenario and device setup are shown in Figure 5a. An HD camera was utilized to scan the Vuforia marker. The camera used is Hewlett‐Packard (HP) w300, featuring a 2‐megapixel COMOS sensor, a maximum frame rate of 30fps, and an image resolution of 1920 × 1080. A male body human phantom (Figure 5b) measuring 45 cm in length, 35 cm in width, and 22 cm in height was used. The phantom surface, made of rubber material that is not easily deformed, is supported by a layer of hard plastic material designed to provide structural support. The CR‐Scan‐Ferret was chosen for 3D body surface imaging. The imaging principle is infrared combined with the depth camera. The working distance ranges from 150 to 700 mm. The maximum range of a single scan is 820 mm×560 mm, with a single‐scan accuracy of 0.1 mm. The single‐frame imaging of the human body phantom is shown in Figure 5c. Additionally, we utilized a high‐precision operating table with three directions of knobs (see Figure 5d) to simulate the treatment couch for position movement. The operating table has a rotation accuracy of 0.02° and a displacement accuracy of 0.01 mm. Regarding translation, a complete rotation of the knob corresponds to a displacement of 0.5 mm on the operating table. With 50 divisions on the knob, each division corresponds to 0.01 mm. For rotation, every full rotation of the knob corresponds to 1°, meaning each division represents 0.02°.

#### 2.2.2 AR‐guided initial position correction

Although AR has the capability for position tracking, the accuracy needs to be improved. Therefore, the AR was utilized solely for monitoring and initial position correction in this study. As shown in Figure 4a, the alignment of the virtual and real coordinate systems was achieved by scanning the real Vuforia marker with the HD camera.

**FIGURE 6:**
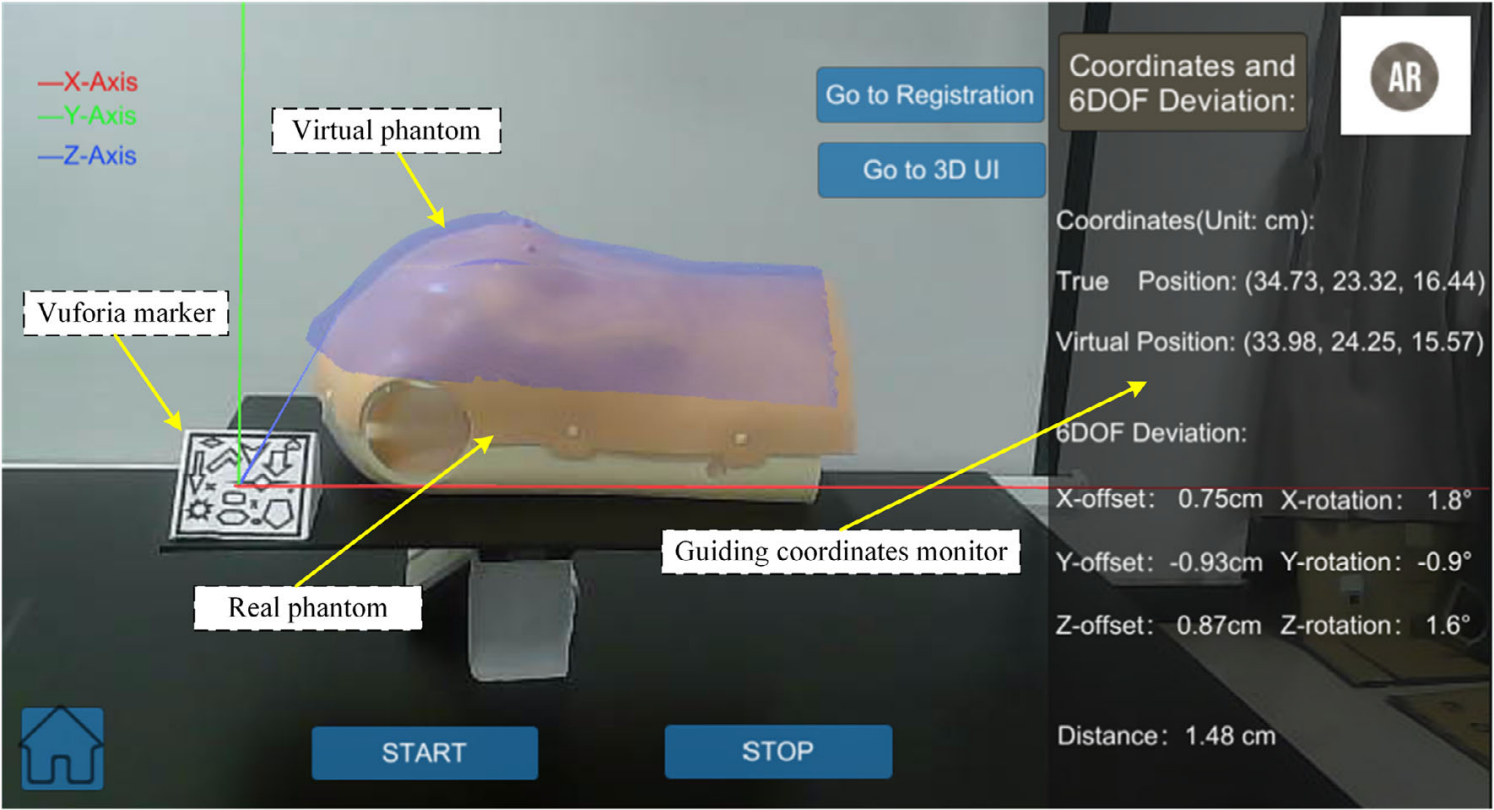
Initial position correction guided by AR and 6DOF deviations.

First, we artificially set an initial position as the target position. The point cloud at the initial locations was captured by CR‐Scan‐Ferret. As shown in Figure 4b,c, the virtual 3D phantom was obtained by CR‐Scan‐Ferret and imported into Unity 3D. Subsequently, the relative position of virtual 3D phantom and the Vuforia markers was binded following the official Vuforia tutorial. The real Vuforia marker was fixed on the table. Secondly, by scanning the real Vuforia marker, the virtual phantom of the initial target position became visible, while the real position phantom remained within the same field of view, as shown in Figure 6. Finally, the real phantom was adjusted by moving it to achieve a substantial overlap between the virtual and real phantom positions. When the displacement offset of each axis was within 1 cm, and the rotation deviation was within 2°, the initial position correction was considered accomplished.

#### 2.2.3 3D surface imaging guided precise PCR

As shown in Figure 4c, after AR‐guided initial position correction, a virtual phantom was obtained at the current position using CR‐Scan‐Ferret. Next, the PCR of this virtual phantom to the initial virtual phantom was executed. Finally, based on the 6 DOF position deviations, operating table was adjusted to complete the positioning process. In this stage, AR solely provided a camera monitoring function. The coordinates and 6 DOF errors shown in the interface were obtained from the PCR result.

The registration error is calculated by comparing the difference between the actual position deviations and the output deviations of PCR. The experimental steps and calculation method are as follows:

Firstly, we designate position A as the target position and perform a CR‐Scan‐Ferret scan at this position to obtain point cloud A. Then, the operating table was moved to define it as position B and another CR‐Scan‐Ferret scan was performed to obtain point cloud B. The actual position 6 DOF deviations between positions B and A were recorded. The rotation deviations in the X, Y, and Z axes were denoted as R_x_, R_y_, and R_z_, and translation deviations as T_x_, T_y_, and T_z_. Next, the proposed PCR method was applied to calculate and output the 6 DOF deviations between the two‐point clouds. The rotation deviations in the X, Y, and Z axes obtained from the registration were denoted as r_x_, r_y_, and r_z_, and the displacement deviations as t_x_, t_y_, and t_z._ Finally, the errors of the proposed system could be obtained by subtracting the actual position deviation from the output deviations of the PCR method.

The rotation errors ΔR could be expressed as:

(3) $$\left\{\begin{array}{c} \;{\Delta R}_{x}=R_{x}\;-r_{x}\\ \;{\Delta R}_{y}=R_{y}\;-r_{y}\\ \;{\Delta R}_{z}=R_{z}\;-r_{z} \end{array}\right.$$

The translation errors ΔT could be expressed as:

(4) $$\left\{\begin{array}{c} \;{\Delta T}_{x}=T_{x}\;-t_{x}\\ \;{\Delta T}_{y}=T_{y}\;-t_{y}\\ \;{\Delta T}_{z}=T_{z}\;-t_{z} \end{array}\right.$$

The distance (D) error at both positions could be expressed as:

(5) $$D\;=\sqrt{{\Delta T}_{x}^{2}+{\Delta T}_{Y}^{2}+{\Delta T}_{Z}^{2}}\;$$

### 2.3 Feasibility evaluation based on the real human body by the proposed system

**FIGURE 7:**
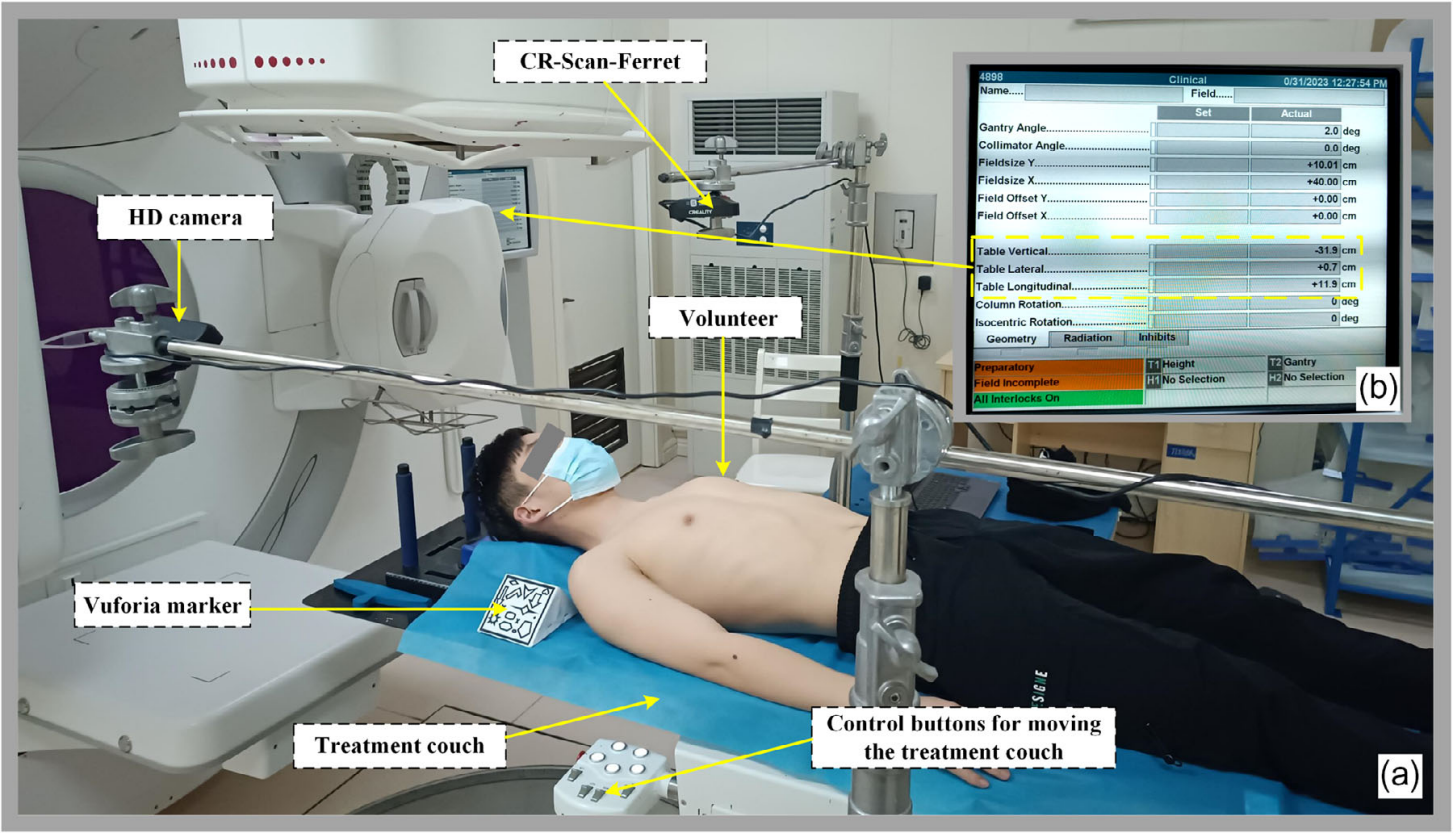
Real human experiment scene of radiotherapy positioning. a: Location of components on the experimental site, b: Accelerator display screen(the each movement of the treatment couch can be read by three values in the yellow dashed box).

To validate the viability of the proposed system and account for patient movement resulting from respiratory factors, positioning experiments were conducted in the radiotherapy room using a real human body. These experiments were divided into breath‐holding tests and free‐breathing tests, with 10 groups for each type tests. The working scenario diagram of the real human body testing was shown in the Figure 7a. Unlike the phantom tests, the research subject in this case was a real human body. Additionally, the operating table was substituted with an authentic radiotherapy couch. Since the couch used exclusively allowed for translation movement, the focus of the tests was solely on studying the translation errors.

In contrast to the phantom tests, the breath‐holding test involved the following steps. Firstly, an initial position was set, and the volunteer was instructed to hold their breath for 5–10 seconds at the end of inspiration. During this breath‐holding period, the surface point cloud was obtained using CR‐Scan‐Ferret. Afterwards, the treatment bed was repositioned, and the movement information could be read and recorded from the accelerator display screen which is shown in the yellow dashed box of Figure 7b. Table Lateral, Table Vertical and Table Longitudinal corresponded to X‐axis, Y‐axis and Z‐axis, respectively. Following the movement, the volunteer was then instructed to hold breath again for 5−10 s at the end of inspiration. During this second breath‐holding period, the surface point cloud was obtained using CR‐Scan‐Ferret. This new point cloud was then registered with the previous point cloud to calculate the registration errors. The free‐breathing tests allowed the volunteer to breathe freely without any intervention and the remaining steps were the same as the breath‐holding experiment.

### 2.4 Interaction envisagement of the phantom‐based CBCT with the proposed system

**FIGURE 8:**
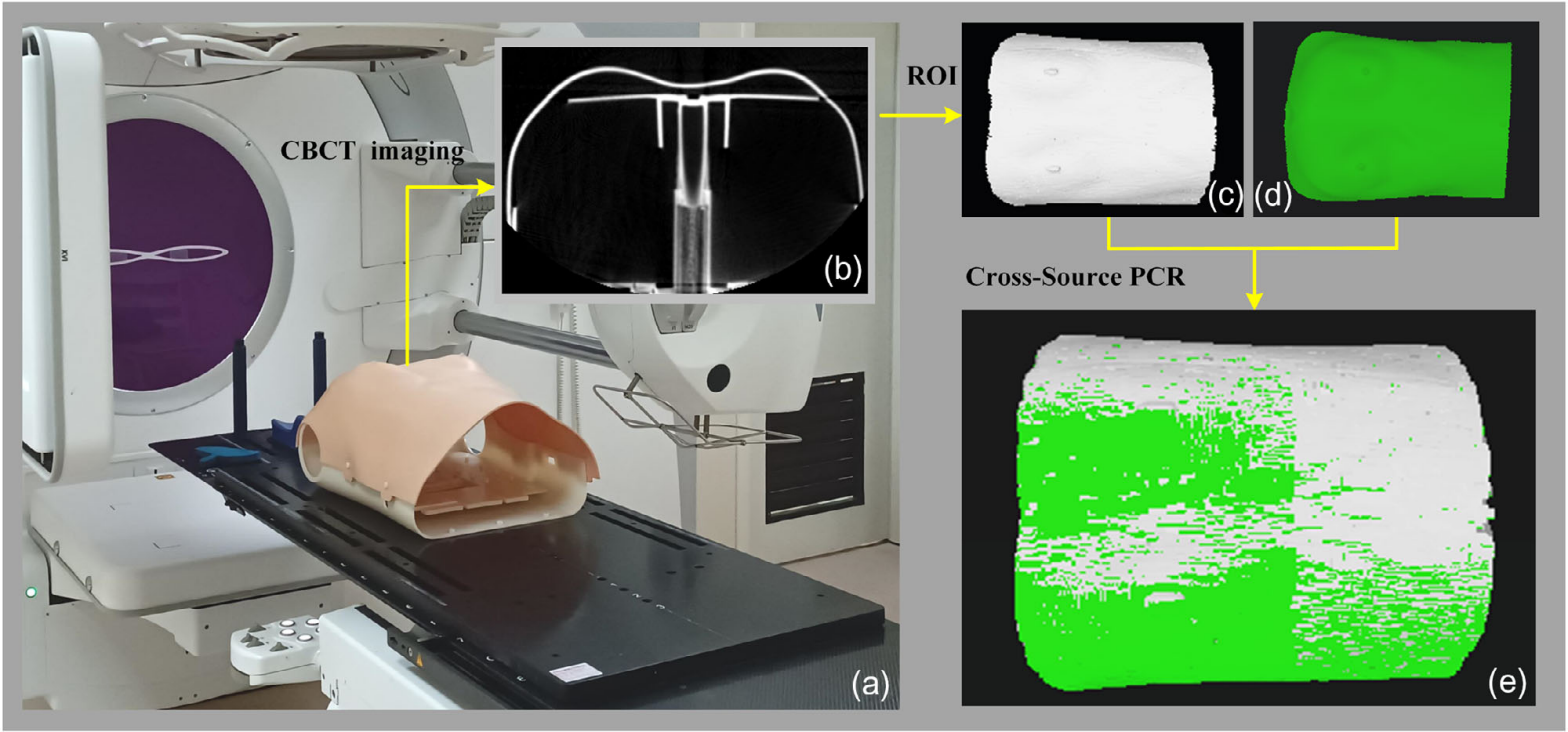
Interaction envisage of the phantom‐based CBCT with the proposed system. a: CBCT imaging scene of the phantom, b: CBCT image of the phantom, c: ROI extraction of surface point cloud of CBCT, d: Surface point cloud of optical scanning, e: CBCT‐optical cross‐source PCR effect.

While the proposed system has only been tested using the phantom and volunteer and has not yet been implemented in the real radiotherapy process, the interaction between the system and CBCT is feasible. To validate this feasibility, we conducted an interactive envisaging study using the phantom as shown in Figure 8. Initially, we performed CBCT imaging on the phantom (see Figure 8a,b). Subsequently, a point cloud of the phantom's surface was segmented and extracted from the CBCT images (Figure 8c). Simultaneously, we employed CR‐Scan‐Ferret for optical surface imaging of the phantom, obtaining an optical surface point cloud (Figure 8d). The CBCT surface point cloud and the optical surface point cloud had the same ROI. Finally, the two‐point clouds were registered using CBCT‐optical cross‐source PCR to achieve the interaction between CBCT and the proposed system (Figure 8e).

## 3 RESULTS

### 3.1 Evaluation of 3D surface reconstruction phantom

**FIGURE 9:**
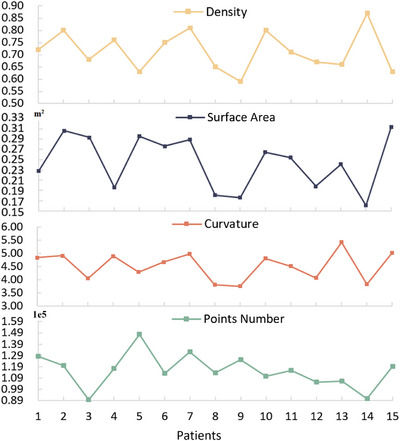
Line plots of point cloud reconstruction evaluation from 15 patients.

We obtained body surface phantoms for 15 patients CT images and performed ROI extraction to demonstrate the applicability of the proposed PCR procedure to different patients. Points number, density, curvature, and surface area were selected to describe point cloud phantoms. The results are presented in Figure 9.

### 3.2 Exploration of the optimal steps of PCR

In this paper, each step of PCR was investigated by comparison tests to identify factors that may affect registration results. The tests included variations in ICP types, down‐sampling methods, filtering types, and sampling ratios, and each test consisted of 50 groups. The results are presented in Table 1.

**TABLE 1:** Exploration of the optimal steps for PCR procedure.

Groups	X (mm)	Y (mm)	Z (mm)	X (°)	Y (°)	Z (°)	D (mm)
Voxel	0.7 ± 0.18	0.85 ± 0.2	1.23 ± 0.98	1.93 ± 0.85	0.97 ± 1.08	1.22 ± 1.19	1.70 ± 0.91
Random	0.46 ± 1.09	0.24 ± 0.16	1.06 ± 0.62	1.49 ± 0.76	1.01 ± 2.86	0.77 ± 1.03	1.32 ± 1.10
Uniform	0.18 ± 0.49	0.16 ± 0.18	0.26 ± 0.62	0.05 ± 0.63	0.17 ± 0.41	0.22 ± 0.62	0.35 ± 0.10
Radius	0.0004 ± 0.0001	0.0002 ± 0.0002	0.0002 ± 0.0002	0.0005 ± 0.0003	0.0003 ± 0.0001	0.0004 ± 0.0001	0.0005 ± 0.0003
Statistics	0.0003 ± 0.0001	0.0002 ± 0.0001	0.0002 ± 0.0002	0.0005 ± 0.0001	0.0002 ± 0.0001	0.0005 ± 0.0001	0.0004 ± 0.0003
ICP types difference	0.17 ± 0.18	0.34 ± 0.29	0.52 ± 0.29	0.50 ± 0.55	0.56 ± 0.43	0.53 ± 0.54	0.72 ± 0.32

*Note*: Voxel/Random/Uniform: Down‐sampling methods, Radius/Statistics: Filtering types, ICP types difference: The difference in registration error between Point‐to‐Plane ICP and Point‐to‐Point ICP.

In the comparison experiment results of ICP types, the 6 DOF errors and D errors of Point‐to‐Plane ICP were smaller than Point‐to‐Point ICP. The D error of Point‐to‐Plane ICP was reduced by 0.71 ± 0.32 mm compared with Point‐to‐Point ICP. The corresponding parameters were then set to ensure a similar number of points after sampling for the three down‐sampling methods. The 6 DOF errors after using uniform down‐sampling were within 1 mm and 1°. While the maximum rotation error after voxel down‐sampling amounted to 1.93 ± 0.85°, and the maximum error of random down‐sampling reached 1.49 ± 0.76°. According to discrete point elimination results, filtering had a negligible effect on the registration results, which were all less than 0.001. We also chose to eliminate the more uniform statistical filtering instead of discarding the discrete point elimination since filtering removed some points bringing a reduction in registration calculations. Based on the above optimal methods, we further explored the effect of sampling ratio on registration efficiency. The results, shown in Table 2, indicated that the time consumption of PCR decreased with an increasing sampling ratio for both Point‐to‐Plane ICP and Point‐to‐Plane ICP. In addition, compared to no sampling, the registration time of Point‐to‐Point ICP decreased from 1.711 to 0.152 seconds at a sampling ratio of 20, and Point‐to‐Plane ICP reduced from 0.437 to 0.111 seconds. Hence, the single registration time of the final PCR method is only 0.111 seconds. The acceleration ratios reached 11.26 and 3.94 for Point‐to‐Point ICP and Point‐to‐Plane ICP, respectively.

**TABLE 2:** Time consumption tests with different sampling ratios.

ICP types	No sampling	2	5	8	10	12	15	18	20
Point‐to‐Point	1.711	0.754	0.371	0.269	0.231	0.191	0.181	0.158	0.152
Point‐to‐Plane	0.437	0.265	0.159	0.140	0.129	0.122	0.119	0.116	0.111

*Note*: Unit: second.

Based on the optimal investigations above, we determined the optimal steps of the proposed PCR procedure, consisting of Point‐to‐Plane ICP, statistical filtering, uniform down‐sampling, and a sampling ratio of 20.

### 3.3 Results of PCR in virtual scene

As shown in Figure 3e, the two‐point cloud phantom positions overlapped, which indicated that the proposed PCR procedure was feasible for positioning medical 3D phantoms. Based on the proposed PCR procedure, we conducted registration tests on 15 patients' phantoms, and each adopted 10 random position settings in virtual scene to simulate the patient's position changes during the radiotherapy. As shown in Table 3, the maximum rotation error and displacement error were both in the Y axis, which was 0.013 ± 0.028 mm and 0.008 ± 0.0214°, respectively, and the D error was only 0.015 ± 0.029 mm.

**TABLE 3:** PCR results for 15 patient phantoms.

Groups	X (mm)	Y (mm)	Z (mm)	X (°)	Y (°)	Z (°)	D (mm)
1	0.001 ± 0.004	0.100 ± 0.316	/	/	0.026 ± 0.084	/	0.101 ± 0.316
2	0.028 ± 0.042	0.042 ± 0.084	0.010 ± 0.032	0.040 ± 0.097	0.080 ± 0.140	/	0.056 ± 0.096
3	/	0.043 ± 0.091	/	/	/	0.060 ± 0.107	0.043 ± 0.091
4	/	/	/	/	/	/	/
5	/	0.001 ± 0.003	/	0.003 ± 0.005	0.001 ± 0.003	0.015 ± 0.029	0.001 ± 0.003
6	0.001 ± 0.003	/	/	0.010 ± 0.030	/	0.011 ± 0.033	0.001 ± 0.003
7	/	/	0.002 ± 0.006	/	0.001 ± 0.004	/	0.002 ± 0.006
8	0.001 ± 0.002	0.001 ± 0.003	/	/	0.002 ± 0.007	/	0.001 ± 0.004
9	/	/	/	0.010 ± 0.033	/	/	/
10	0.001 ± 0.003	0.001 ± 0.004	0.005 ± 0.016	/	0.011 ± 0.035	0.001 ± 0.004	0.006 ± 0.016
11	0.001 ± 0.002	/	0.001 ± 0.002	/	/	/	0.001 ± 0.003
12	/	/	/	/	/	/	/
13	/	/	/	0.006 ± 0.02	0.005 ± 0.015	0.012 ± 0.039	/
14	/	/	/	/	/	/	/
15	0.007 ± 0.021	/	0.001 ± 0.002	/	/	/	0.007 ± 0.021
Mean	0.003 ± 0.007	0.013 ± 0.028	0.001 ± 0.003	0.005 ± 0.01	0.008 ± 0.021	0.007 ± 0.016	0.015 ± 0.029

*Note*: /: errors are less than 0.001.

### 3.4 Results of phantom tests by AR‐and‐PCR‐guided positioning system

10 phantom experiments were utilized for feasibility verification of the proposed system. As shown in Table 4, the translation errors in each direction and the D error were at the sub‐millimeter level. The average D error was 0.6 ± 0.2 mm. Moreover, the displacement errors were 0.41 ± 0.2 mm, 0.26 ± 0.16 mm, and 0.27 ± 0.2 mm in the X, Y, and Z axes. The angle deviations were 0.18 ± 0.08°, 0.33 ± 0.13°, and 0.29 ± 0.23°in the X, Y, and Z axes.

**TABLE 4:** Registration results of human body phantoms simulating radiotherapy positioning guided by proposed system.

Groups	X (mm)	Y (mm)	Z (mm)	X (°)	Y (°)	Z (°)	D (mm)
1	0.13	0.17	0.06	0.28	0.24	0.19	0.22
2	0.46	0.23	0.49	0.11	0.56	0.08	0.71
3	0.25	0.33	0.21	0.13	0.11	0.06	0.46
4	0.17	0.15	0.23	0.12	0.23	0.15	0.32
5	0.29	0.55	0.08	0.26	0.31	0.03	0.61
6	0.58	0.32	0.45	0.27	0.43	0.61	0.80
7	0.75	0.12	0.02	0.20	0.44	0.62	0.76
8	0.34	0.34	0.52	0.15	0.24	0.25	0.71
9	0.54	0.01	0.48	0.04	0.44	0.51	0.72
10	0.58	0.42	0.13	0.19	0.32	0.43	0.73
Mean ± Std	0.41 ± 0.20	0.26 ± 0.16	0.27 ± 0.20	0.18 ± 0.08	0.33 ± 0.13	0.29 ± 0.23	0.60 ± 0.20

### 3.5 Results of real human body tests by AR‐and‐PCR‐guided positioning system

As shown in Table 5, the average accuracy of breath‐holding positioning test was still at the sub‐millimeter level, where the average error of the X, Y and Z axes were 0.59 ± 0.12 mm, 0.54 ± 0.12 mm, and 0.52 ± 0.09 mm, and the average D error was 0.96 ± 0.22 mm. The result of free‐breathing positioning test is shown in Table 6. The average errors of X, Y, and Z axes were still less than 1 mm, respectively 0.86 ± 0.21 mm, 0.78 ± 0.19 mm, 0.68 ± 0.19 mm. Although the mean D error was greater than 1 mm, it still falls within a clinically acceptable error margin.

**TABLE 5:** Results of breath‐holding positioning test based on the real human guided by proposed system.

Groups	X (mm)	Y (mm)	Z (mm)	D (mm)
1	0.66	0.41	0.44	0.80
2	0.61	0.49	0.66	1.05
3	0.57	0.51	0.57	0.91
4	0.48	0.65	0.48	0.88
5	0.66	0.56	0.49	0.99
6	0.82	0.69	0.54	1.44
7	0.44	0.66	0.45	0.83
8	0.48	0.45	0.62	0.82
9	0.53	0.51	0.38	0.69
10	0.67	0.66	0.57	1.21
Mean ± Std	0.59 ± 0.12	0.54 ± 0.10	0.52 ± 0.09	0.96 ± 0.22

**TABLE 6:** Results of free‐breathing positioning test based on the real human guided by proposed system.

Groups	X (mm)	Y (mm)	Z (mm)	D (mm)
1	1.23	0.83	0.58	2.54
2	0.81	0.91	0.56	1.80
3	0.66	0.91	0.84	1.97
4	0.62	0.75	1.11	2.18
5	1.17	0.52	0.66	2.07
6	0.88	0.89	0.49	1.81
7	0.76	0.68	0.78	1.65
8	0.67	1.07	0.59	1.94
9	0.97	0.82	0.71	2.12
10	0.87	0.42	0.51	1.19
Mean ± Std	0.86 ± 0.21	0.78 ± 0.19	0.68 ± 0.19	1.93 ± 0.36

## 4 DISCUSSION

In this paper, a high‐precision radiotherapy positioning system is developed by combining 3D surface reconstruction, PCR, and AR interaction technologies, which can improve the AR guidance stability and the radiotherapy positioning accuracy.

The system accuracy is mainly ensured by the PCR procedure. The high accuracy required for radiotherapy positioning makes it necessary to fully explore the optimal steps of PCR. Comparison experiments were designed to observe position offset errors. The results demonstrated that factors including the down‐sampling method, the down‐sampling ratio, and the ICP type could influence registration precision. First, the speed and accuracy of Point‐to‐Plane ICP were significantly better than Point‐to‐Point ICP. Previous studies^15^ showed that Point‐to‐Plane ICP had a wider convergence range and higher convergence speed when finding adjacent points, which was also consistent with our results. Second, among the three down‐sampling methods, uniform down‐sampling is more suitable for registration for positioning. The reason may be that the uniform down‐sampling only reduces point cloud density and does not affect the phantom size. Besides, the reduction of points decreases the calculation time of PCR.^9^ These two features provide guarantees for accuracy and speed of the proposed system. Finally, as the down‐sampling ratio increases, the registration time decreases while accuracy is also not reduced. The reason is that the number of points traversed is diminished and the ICP algorithm itself is more suitable for sparse point clouds.^16^, ^17^ In addition, the 15 patients' results of point cloud reconstruction and PCR indicate that the proposed PCR is suitable for different patients. AR and PCR are the two key techniques used in the proposed system. The ICP registration depends on the initial position of the point cloud. Otherwise, it may easily be trapped in local optima.^18^ In this study, AR‐guided initial position correction compensates for this drawback of ICP, which ensures the accuracy of the proposed workflow. Compared with other radiotherapy positioning methods, the proposed workflow thus achieves contact‐less, marker‐less, and high‐precision radiotherapy positioning. The traditional manual positioning methods^19^ are time‐consuming and have low precision. Patient positioning using laser and skin surface markers is also difficult to meet the demand for more accurate patient positioning.^20^ Image‐guided radiotherapy such as CBCT and MRI can improve patient positioning accuracy, but they come with obvious drawbacks. For instance, it will bring more complex workflows to radiotherapy technicians and additional radiation to patients. Most importantly, the positioning error can only be reflected by these technologies at the time of scanning, while real‐time guidance cannot be achieved.^6^ By contrast, SGRT can achieve radiation‐free and high‐precision radiotherapy placement. Nevertheless, it has a single function, a finite field of view, and boundedness in human‐computer interaction integrating and multidimensional information.^24^

Recently contact‐less and marker‐less medical research using AR have become popular. AR interaction using HMD can provide users with a better AR immersive experience. However, it is more suitable for applications that do not require high stability and accuracy, including medical AR games,^22^ medical teaching tools,^23^ simulation experiments,^24^ etc., since users can easily get fatigued when using HMD. Additionally, it is difficult to ensure system stability due to the wearer's angle, height, etc., and only relatively steady results can be recorded.^6^ Therefore, to meet the accuracy and stability requirements of radiotherapy positioning, we used an HD camera instead of an HMD, which can provide more stable recognition results and also enables AR surveillance. Compared with other studies using AR,^6^, ^21^, ^25^ the error of the proposed system is significantly lower, especially the displacement deviations in sub‐millimeter, which is comparable to the advanced SGRT.^26^

Although the application of PCR has become increasingly extensive in the medical field,^27^, ^28^, ^29^ few studies focus on its optimal utilization in radiotherapy. Some studies^21^, ^25^ have employed ICP registration for radiotherapy positioning, similar to our approach. Nevertheless, the difference lies in our further exploration of the application of ICP, including investigating the sampling method, discrete point elimination, ICP type, etc., to enhance the accuracy and stability of registration in medical phantoms. In similar studies using Point‐to‐Plane ICP for radiotherapy positioning,^21^ the optimal positioning error for human thoracic and abdominal phantom was still above 1 mm, while that in our phantom study was in sub‐millimeter, significantly improving the accuracy, which further confirmed the necessity of thoroughly exploring the steps of PCR. Furthermore, the optimal steps of PCR may be different under different clinical situations, which requires us to explore the balance between accuracy and speed by combining different clinical situations.

The experiments conducted on real human body have demonstrated the feasibility of the proposed method in the real radiotherapy room environment and with the actual human body. During the tests, we also explored the impact of respiration on the precision. The results indicate that the accuracy of positioning during breath‐holding tests remains at the sub‐millimeter level, albeit slightly lower than in the phantom experiments. This discrepancy may arise from difference between the radiotherapy room environment and the laboratory environment, such as light, the shape and color difference between the phantom and the real human body, difference of the operation table and the real treatment bed, etc. Furthermore, the accuracy of positioning during free‐breathing tests is lower than that of breath‐holding tests. This discrepancy can be attributed to the changes in body surface shape caused by breathing, which increases the dissimilarity between the two scanned point clouds and leads to higher registration errors. However, despite the decreased accuracy observed in free‐breathing positioning, the results still fall within the clinically acceptable error range and are higher than those reported in similar studies.^6^, ^21^, ^25^ Nevertheless, the decrease in the accuracy highlights the need to minimize the effects of breathing‐induced deformation during positioning. To address this, it is necessary to optimize the registration algorithm, such as by incorporating a deformation registration step to correct for breathing errors. In addition to considering the impact of breathing. other sources of error should also be taken into account and overcome. Firstly, since the CR‐Scan‐Ferret used is an optical imaging tool, the imaging accuracy may be affected by environmental factors, such as light conditions, patient skin color and so on. Secondly, the performance limitation of CR‐Scan‐Ferret can introduce imaging errors that subsequently affect the registration results. Moreover, in phantom tests, manual movements of the operating table can introduce some errors. Lastly, in the tests conducted on actual human subjects, it is challenging to ensure that the volunteer remain in the same state during the breath‐holding stage, which could have an impact on the results.

The proposed system offers several advantages for radiotherapy positioning. Firstly, the coordinates and 6 DOF errors on AR interface can be utilized for monitoring and adjusting during the positioning process. Secondly, the combination of AR and PCR ensures the accuracy of the proposed system and a more intuitive and direct positioning process. Finally, the reduced use of CBCT helps to lower non‐treatment doses and treatment costs for patients. Additionally, the proposed system can be integrated with other technologies such as structured light, laser and other imaging methods to achieve real‐time patient positioning and mitigate human errors. In addition, although real‐time positioning was not achieved, the high accuracy achieved indicates promising prospects for the development of the proposed system. And the remarkably fast single registration time of only 0.111 seconds provides a solid foundation for future real‐time radiotherapy positioning guidance. Finally, the phantom‐based interaction envisage between CBCT and the proposed system also provides a feasibility for its application in future real patients.

The proposed system is expected to achieve high‐precision radiotherapy positioning without contact. However, it is still in the development stage and has not been applied in clinical practice due to certain limitations.

Firstly, the location settings of CR‐Scan‐ferret limit the potential application scenarios. However, in future developments, two CR‐Scan‐ferret could be placed on either side of the patient, enabling the system to be available for the real radiotherapy process. Secondly, the interactions between CBCT and the proposed system were only based on the phantom since human experiments would involve complex and time‐consuming ethical arguments. We will conduct the interaction between CBCT of real patients and the proposed system in the future. Additionally, CBCT contains the correspondence of internal organ and tumor of patients and the body surface. Thus, we will use CBCT‐optical surface cross‐source registration to find correlations between optical surface point clouds and anatomical information in patients in the future. Thirdly, the test point cloud phantoms used are offline reconstructed due to the CR‐Scan‐Ferret, which causes inability to achieve real‐time registration. We will focus on improving the speed of surface imaging and registration to enable real‐time registration. Fourthly, the single HD camera limited the AR field of view. Thus, the use of multiple cameras will be explored to achieve multi‐angle AR visualization, which enhances the AR experience and expands the field of view for AR monitoring.

## 5 CONCLUSION

In this paper, an AR‐and‐PCR‐guided system was developed for marker‐less radiotherapy positioning. The proposed method achieves registration accuracy at the sub‐millimeter level in phantom experiments. In real human tests, the effect of respiration on the proposed system was explored, confirming that although the system is affected by respiration, it still provides relatively high accuracy, and the accuracy remains within a clinically acceptable range. While the system is not applied to actual patients, it demonstrates several advantages including high registration accuracy and speed, stable position verification results, and stable AR tracking. The system thus holds significant potential for further clinical application of radiotherapy.

## AUTHOR CONTRIBUTIONS

All authors contributed to the study conception and design. Data collection was performed by Ziwen Wei, Xiaolong Wu and Ligang Xing. Shaozhuang Zhai and Junchao Qian analyzed and Interpreted data for the work. The first draft of the manuscript was wrote by Shaozhuang Zhai. Final approval of the work to be published was finished by Junchao Qian.

## ACKNOWLEGMENTS

This work was supported by the National Natural Science Foundation of China (grant number U1932158, 81871085 and 82271519), Natural Science Foundation of Shandong Province (grant number ZR2019LZL018), Anhui Province Funds for Distinguished Young Scientists (grant number 2208085J10), Collaborative Innovation Program of Hefei Science Center, CAS (grant number 2019HSC‐CIP003), China Postdoctoral Science Foundation (grant number 2019M652403), Project of Postdoctoral Innovation of Shandong Province (grant number 202002048).

## CONFLICT OF INTEREST STATEMENT

The authors declare no conflicts of interest.
